# The validity of using one force platform to quantify whole-body forces, velocities, and power during a plyometric push-up

**DOI:** 10.1186/s13102-021-00330-z

**Published:** 2021-08-30

**Authors:** Zhanxin Sha, Boyi Dai

**Affiliations:** 1 School of Kinesiology and Nutrition, College of Education and Human Sciences, The University of Southern Mississippi, Hattiesburg, MS USA; 2 Division of Kinesiology and Health, University of Wyoming, Laramie, WY USA

**Keywords:** Upper body, Strength, Assessment

## Abstract

**Background:**

Previous studies have typically measured velocity and power parameters during the push-up, either using one or two force platforms. The purpose of the study was to compare the force, velocity, and power parameters between the one-force-platform method and the two-force-platform method during plyometric push-ups.

**Methods:**

Thirty-four physically active young adults participated in the study to perform the plyometric push-up. For the two-force-platform calculation method, the forces applied to the feet and hands were both measured. For the one-force-platform calculation method, the forces applied to the feet were assumed to be constant, while the forces applied to hands were measured by one force platform. Whole-body linear velocities were calculated based on the impulse and momentum theorem. Whole-body power was calculated as the product of the whole-body forces and velocities.

**Results:**

The one-force-platform method overestimated the whole-body velocities and power compared with the two-force-platform method (1.39 ± 0.37 m/s vs. 0.90 ± 0.23 m/s, Cohen’s d = 1.59, p < 0.05; 1.63 ± 0.47 W/body weight vs. 1.03 ± 0.29 W/body weight, Cohen’s d = 1.49, p < 0.05). These differences were caused by the decreased forces applied to the feet compared to the initial value throughout most of the push-up phase. Large to perfect correlations (r = 0.55 – 0.99) were found for most variables between the two-force-platform and one-force-platform methods. Previous findings of push-up velocities and power using the two-force-platform and one-force-platform methods should be compared with caution. While the two-force-platform method is recommended, linear regression equations may be used to predict velocities and power parameters obtained from one force platform.

**Conclusions:**

For those professionals who need to accurately quantify kinetic variables during the plyometric push-up, the two-force-platform method should be considered.

## Introduction

Muscular strength assessments are essential components in many research studies and practical settings. Muscular strength assessments allow exercise scientists and strength conditioning coaches to identify strengths and weaknesses for establishing specific training goals [1]. Push-up exercises represent a popular strategy for evaluating upper-extremity strength training, rehabilitation, and muscular endurance. Push-up exercises are also widely used for clinical rehabilitation purposes [1].

Push-up could be modified and adapted to match different training goals [2–19]. Training with traditional and plyometric push-ups could increase upper body strength [17]. Previous studies have investigated neuromuscular activation pattern, force, and velocity, parameters during different push-up exercises. Several studies have examined patterns of muscle activation during push-ups with various hand and foot placements [2–12]. Greater muscle activation was observed with hands in a narrow base position [3]. Suspension training systems and certain unstable surfaces were likely to elicit high levels of muscle activation [4–12]. Previous studies [13–18] have also assessed the rate of force development, peak vertical ground reaction forces (GRFs), and impulse during different push-up variations. Push-ups with feet elevated produced a higher peak vertical GRF than knee flexed positions and hands elevated push-up variations [13–18]. Compare with traditional push-ups, the peak vertical GRF and rate of force development during takeoff were greater in plyometric countermovement push-ups [14, 15]. In these previous studies, force parameters (rate of force development, peak force, impulse) have been typically obtained using a force platform that measures the GRFs applied on the arms [12–19]

Power is another kinetic parameter to quantify the intensity and performance of an exercise. Previous studies [20–23] have quantified the power performance of the lower extremities during vertical jumps by measuring the GRFs applied to the legs. However, there appears to be no consensus on how power should be calculated from a single force platform or multiple force platforms during push-ups [13–15, 17, 24]. In a push-up, both the hands and feet experience GRFs, and the total GRFs should be the sum of both components [13]. When only one force platform under the hands is used, the assumption of constant forces being applied to the feet must be made to calculate the total GRFs [24]. Therefore, previous researchers [13–15] indicated that power might not be accurately measured from one force platform. Hinshaw et al. [17] adapted two synchronized force platforms to investigate power performance during push variations. The authors measured forces applied to both hands and feet to calculate whole-body velocities based on the impulse and momentum theorem to calculate power. However, whether the magnitude of force at feet during the push-up is relatively constant and how it would influence the whole-body force, velocity, and power parameters during push-ups is still unclear.

Plyometric push-ups involve the utilization of fast eccentric loading to produce increased concentric forces through the stretch–shortening cycle. The plyometric push-up resulted in significantly greater improvements in medicine ball throwing and peak vertical GRF than the traditional push-up [14–16, 25, 26]. However, there was a paucity of studies to quantify power output during plyometric push-ups [15]. Based on the literature, one study [17] adapted two force platforms, and the other studies used one force platform to quantify push-up power [14–16, 25, 26]. The discrepancies of power values among previous calculation methods have not been quantified.

An accurate assessment of the training volume and intensity is a crucial aspect of resistance exercises [27]. Good strength and power assessments need to be reliable, valid, and objective [1]. The push-up exercise is extensively employed in rehabilitation and strength and conditioning programs [1, 27]. Previous research applied different methods to quantify kinetic outcomes of push-ups, but the “golden standard” calculation method has yet been established [27]. While using one force platform may create convenience for data collection, its validity in calculating mechanical variables compared to two force platforms needs to be determined. Therefore, the purpose of the current study was to compare the force, velocity, and power parameters between the one-force-platform and the two-force-platform method during plyometric push-ups. It was hypothesized that the two-force-platform and one-force-platform methods would demonstrate significant differences in whole-body force, velocity, and power parameters due to the non-constant force applied to the feet.

## Method

### Subject

The current study performed a secondary analysis of previously collected data, in which the push-up power was compared among several push-up variations [17]. However, this previous study only used the two-force-platform method without comparing the accuracies between the one-force-platform and two-force-platform methods during plyometric push-ups. A total of 17 male and 17 female physically active young adults with an age of 18 years or older (age: 21.9 ± 3.5 years; mass: 70.2 ± 13.5 kg; height: 1.74 ± 0.10 m) participated. To be eligible for the study, each participant needed to participate in exercises or sports activities at least three times per week and had experience in performing push-up exercises for training. Individuals were excluded if they (1) had a major upper extremity injury that involved surgical treatment, (2) had an upper extremity injury that prevented participation in physical activity for more than 2 weeks over the previous 6 months, or (3) possessed any other conditions that prevented them from participating at maximal effort activities. The current study was approved by the XXX Institutional Review Board. Participants signed informed consent forms prior to participation.

### Procedure

Participants wore athletic attire and standard running shoes (Ghost5, Brooks Sports, Bothell, Washington). All Participants conducted a warm-up protocol, including five-minute running with self-selected speed on a treadmill and self-selected dynamic stretching of the upper body. Two force platforms (FP4060-05-PT, Bertec Corp, Columbus, OH) were used to collect vertical GRFs applied to the hands and feet, respectively. These two force platforms were synchronized at a sampling frequency of 1000 Hz by the Digital Acquire 4.12 software (Bertec Corp, Columbus, OH). Each participant had one practice trial prior to official data collection. Each participant completed three official trials.

**Fig. 1:**
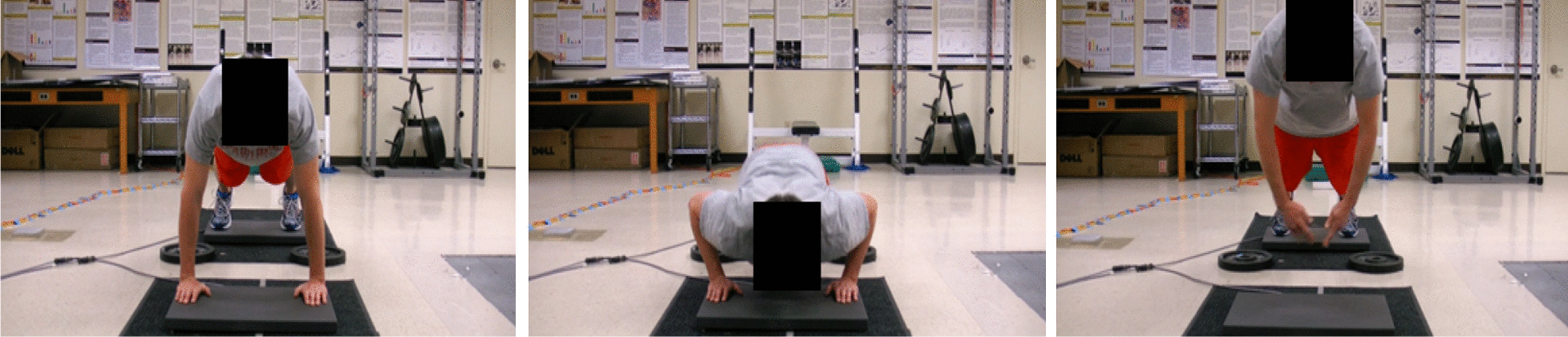
The plyometric push-up on two force platforms

For the plyometric push-up, at the starting position, participants positioned their hands right below their shoulders with feet shoulder-width apart (Fig. 1). When instructed, participants lowered their elbows to the height of their shoulders and pushed up as fast and as forcefully as they could with the encouragement to propel the upper body as high as possible from the force platform while keeping their feet on the force platform [17, 28]. However, the exact elbow flexion, shoulder flexion, or shoulder abduction angles were not controlled. A trial was discarded and repeated when a participant did not lower their elbows to their shoulder height as visually inspected by the researchers. Participants had a minimum of two-minute rest between each trial to minimize fatigue effects.

### Data reduction

Vertical GRFs were extracted and filtered with a 100-Hz low-pass Butterworth filter for future analyses. Higher cut-off frequencies were examined but demonstrated minimal differences. Two methods (two-force-platform and one-force-platform) were used to calculate whole-body forces, velocities, and power. For the two-force-platform method, the GRFs applied to the feet and hands were both measured throughout the push-up.

The beginning of the push-up was defined as the force becoming 15 N lower than the GRFs at hands. The end of the push-up phase was defined as when the force was lower than 15 N. The Trapezoidal Rule was applied to calculate the integral of the force–time curve to calculate linear impulse. Whole-body linear velocity was calculated based on the impulse and momentum theorem.

$${\int }_{{t}_{start}}^{{t}_{end}}\left(\mathrm{GRFs} at hands+\mathrm{GRFs} at feet-m*g\right)dt=m*v$$

Next, whole-body power was calculated as the product of the whole-body force and velocity.

$$\mathrm{Power}=\left(\mathrm{GRFs} at hands+\mathrm{GRFs }at feet\right)* v$$

The same algorithm was applied to the one-force-platform method, except that the GRFs applied to the feet were calculated as body weight minus the GRFs applied to the hands at the starting position. The GRFs applied to the feet were then assumed to be this constant number throughout the push-up, while the GRFs applied to the hands were measured by one force platform.

A total of eight variables were extracted from both calculation methods, including GRFs applied to the hands, peak whole-body GRFs, peak whole-body velocity, peak whole-body power, GRFs applied to the feet at the starting position, mean GRFs applied to the feet during the push-up (from start to end), whole-body GRFs at the peak whole-body power, and whole-body velocity at the peak whole-body power. GRFs and power were normalized to body weight.

### Statistical analysis

Data normality was assessed using the Shapiro–Wilk Test. Paired t-tests were performed to identify the differences in dependent variables between the two-force-platform and one-force-platform methods. Independent t-tests were performed to determine the sex differences in kinetic dependent variables. Cohen’s d effect size with 95% confidence intervals calculated. The magnitude of the effect size was interpreted as suggested by Cohen [29]: 0.0 to 0.19–trivial; 0.20 to 0.49–small; 0.50 to 0.79–moderate; > 0.80–large. Spearman's correlations and simple regression were performed between the two-force-platform and one-force-platform methods for each dependent variable. Statistical significance for all the statistical tests was set at p ≤ 0.05. The strength of correlations was defined as: minor correlation (0.10 < r ≤ 0.30); moderate correlation (0.30 < r ≤ 0.50); large correlation (0.50 < r ≤ 0.70); very large correlation (0.70 < r ≤ 0.90); and perfect correlation (0.90 < r ≤ 1) [30]. All statistical analyses were completed using the SPSS software 21.0.

## Results

**Fig. 2:**
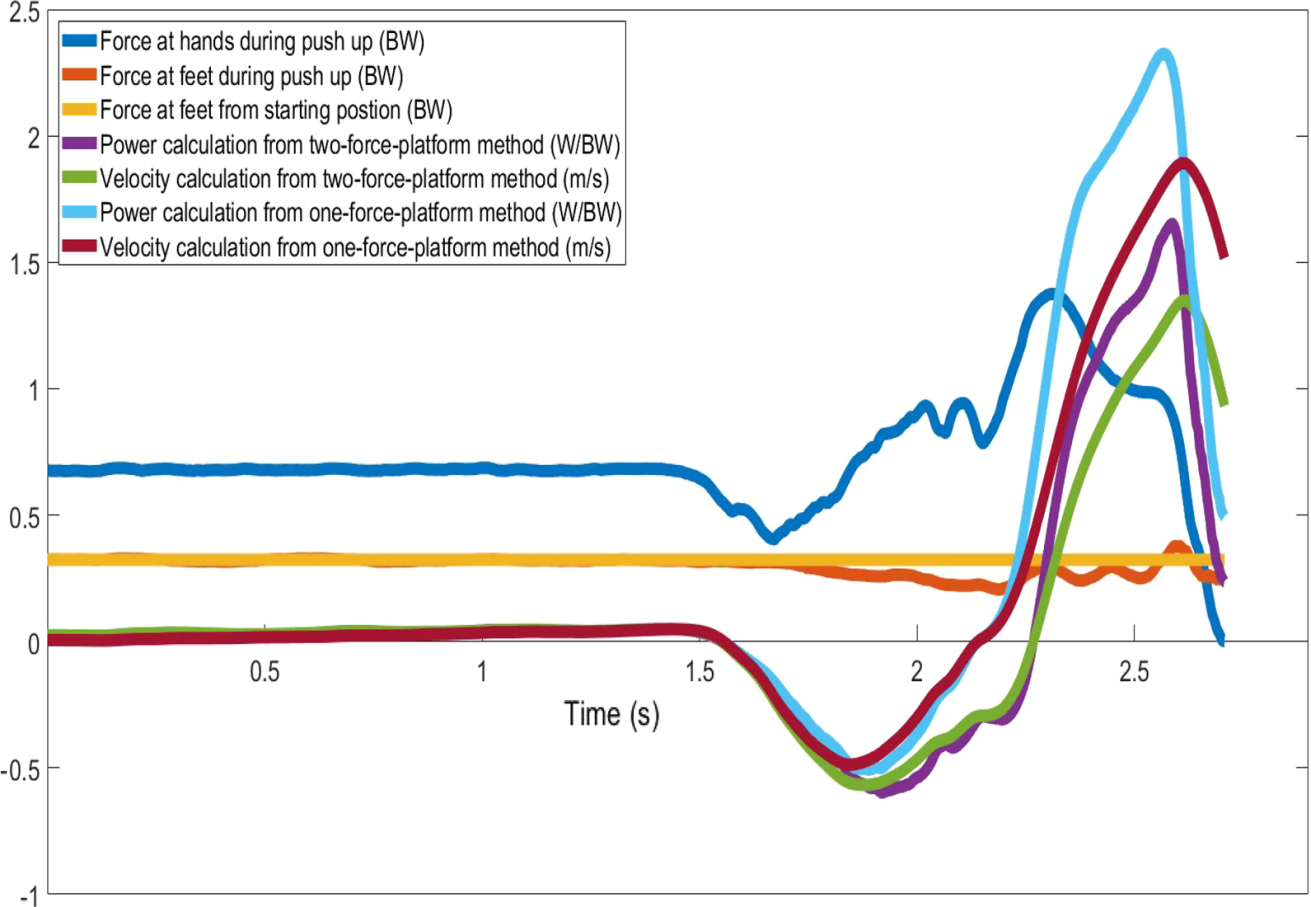
An example of the time-series plot of the force, velocity, and power parameters using the two-force-platform and one-force-platform methods

Data were normally distributed based on the Shapiro–Wilk Test. Paired t-tests showed that the one-force-platform calculation method resulted in greater dependable variables compared with the two-force-platform method. Statistically significant differences were detected in peak whole-body GRFs (1.44 ± 0.21 vs. 1.46 ± 0.22 body weight, p < 0.05), peak whole-body velocities (0.90 ± 0.23 m/s vs. 1.39 ± 0.37 m/s, p < 0.05), peak whole-body power (1.03 ± 0.29 W/body weight vs. 1.63 ± 0.47 W/body weight, p < 0.05), mean GRFs applied to the feet throughout the push-up (0.30 ± 0.04 body weight vs. 0.34 ± 0.04 body weight, p < 0.05), and whole-body velocities at the peak whole-body power (0.85 ± 0.22 m/s vs. 1.35 ± 0.36 m/s,p < 0.05) (Table 1). Between the two-force-platform and one-force-platform methods, no statistically significant difference was detected in whole-body GRFs at peak whole-body power (1.18 ± 0.06 body weight vs. 1.19 ± 0.05 body weight, p > 0.05). The results of Cohen’s d were consistent with the 95% confidence intervals for the mean differences of dependent variables and p values of the t-tests, with most being large effect sizes (Table 1). The comparisons which demonstrated non-significant p values also showed small effect sizes. An example of the time-series plot of the push-up force, velocity, and power parameters using the two-force-platform and one-force-platform methods was illustrated (Fig. 2).

**Table 1:** Descriptive data, statistical comparisons, and effect sizes between the two-force-platform and one-force-platform methods

	Two-force-platforms method	One-force-platform method	Confidence interval and statistical significance	Effect sizes (Cohen’s d)
Peak GRFs applied to hands (body weight)	1.12 ± 0.23	1.12 ± 0.23	– –	– –
Peak whole-body GRFs (body weight)	1.44 ± 0.21	1.46 ± 0.22	[− 0.03 to − 0.01] (p < 0.05)	0.09
Peak whole-body velocities (m/s)	0.90 ± 0.23	1.39 ± 0.37	[− 0.59 to − 0.39] (p < 0.05)	1.59
Peak whole-body power (watt/body weight)	1.03 ± 0.29	1.63 ± 0.47	[− 0.71 to − 0.46] (p < 0.05)	1.49
GRFs applied to the feet at the starting position (body weight)	0.34 ± 0.04	0.34 ± 0.04	– –	– –
Mean GRFs applied to the feet during the push-up (body weight)	0.30 ± 0.04	0.34 ± 0.04	[− 0.04 to − 0.03] (p < 0.05)	0.93
Whole-body GRFs at peak whole-body power (body weight)	1.18 ± 0.06	1.19 ± 0.05	[− 0.02 to 0.00] (p > 0.05)	0.18
Whole-body velocities at peak whole-body power (m/s)	0.85 ± 0.22	1.35 ± 0.36	[− 0.58 to − 0.38] (p < 0.05)	1.61

**Fig. 3:**
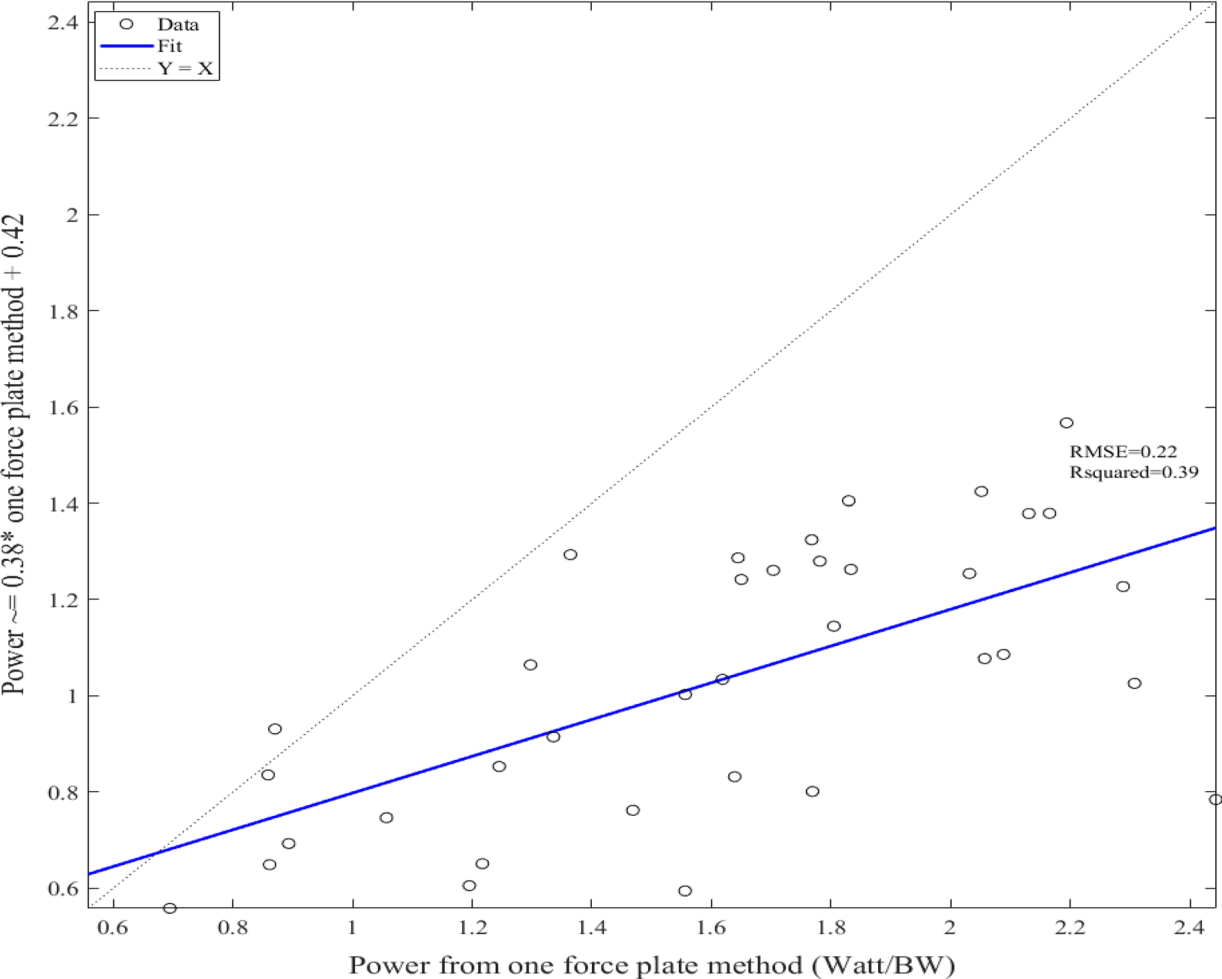
Linear regression to predict the two-force-platform peak power from the one-force-platform power

Perfect correlations (r > 0.90) were observed for peak whole-body GRFs, and average GRFs applied to the feet throughout the push-up between the two-force-platform and one-force-platform calculation methods, while whole-body velocities at peak whole-body power, peak whole-body velocities, and peak whole-body power demonstrate large correlations and whole-body GRFs at peak whole-body power demonstrate very large correlations (Table 2, Fig. 3).

**Table 2:** Correlation and simple regression to predict the two-force-platform variables from the one-platform variables

		Correlation	Simple regression
Peak GRFs applied to hands (body weight)		– –	– –
Peak whole-body GRFs (body weight)		r = 0.99 (p < 0.05)	y = 0.97x + 0.20 r^2^ = 0.97
Peak whole-body velocities (m/s)		r = 0.60 (p < 0.05)	y = 0.37x + 0.39 r^2^ = 0.35
Peak whole-body power (watt/body weight)		r = 0.63 (p < 0.05)	y = 0.38x + 0.42 r^2^ = 0.39
GRFs applied to the feet at the starting position (body weight)		– –	– –
Mean GRFs applied to the feet during the push-up (body weight)		r = 0.92 (p < 0.05)	y = 0.93x – 0.01 r^2^ = 0.84
Whole-body GRFs at peak whole-body power (body weight)		r = 0.72 (p < 0.05)	y = 0.74x + 0.30 r^2^ = 0.50
Whole-body velocities at peak whole-body power (m/s)		r = 0.59 (p < 0.05)	y = 0.38x + 0.38 r^2^ = 0.34

**Table 3:** Descriptive data, statistical comparisons, and effect sizes between male and female from two-force-platform method

	Male	Female	Confidence interval and statistical significance	Effect sizes (Cohen’s d)
Peak GRFs applied to hands (body weight)	1.29 ± 0.17	0.95 ± 0.11	[0.25 to 0.44] (p < 0.05)	2.25
Peak whole-body GRFs (body weight)	1.58 ± 0.18	1.28 ± 0.11	[0.20 to 0.41] (p < 0.05)	2.01
Peak whole-body velocities (m/s)	1.07 ± 0.11	0.72 ± 0.16	[0.25 to 0.45] (p < 0.05)	2.54
Peak whole-body power (watt/body weight)	1.24 ± 0.16	0.81 ± 0.20	[0.31 to 0.56] (p < 0.05)	2.37
GRFs applied to the feet at the starting position (body weight)	0.33 ± 0.03	0.36 ± 0.04	[− 0.06 to − 0.01] (p < 0.05)	0.84
Mean GRFs applied to the feet during the push-up (body weight)	0.28 ± 0.03	0.33 ± 0.04	[− 0.07 to − 0.02] (p < 0.05)	1.41
Whole-body GRFs at peak whole-body power (body weight)	1.24 ± 0.05	1.16 ± 0.05	[0.01 to 0.08] (p < 0.05)	1.60
Whole-body velocities at peak whole-body power (m/s)	1.03 ± 0.11	0.69 ± 0.15	[0.26 to 0.43] (p < 0.05)	2.57

Regarding sex differences identified by independent t-tests, significant differences were detected between male and female in peak GRFs applied to hands (1.29 ± 0.17 vs. 0.95 ± 0.11 body weight, p < 0.05), peak whole-body GRFs (1.58 ± 0.18 body weight vs.1.28 ± 0.11 body weight, p < 0.05), peak whole-body velocities (1.07 ± 0.11 m/s vs. 0.72 ± 0.16 m/s, p < 0.05), peak whole-body power (1.24 ± 0.16 W/body weight vs. 0.81 ± 0.20 W/body weight, p < 0.05), whole-body GRFs at peak whole-body power (1.24 ± 0.05 body weight vs.1.16 ± 0.05 body weight, p < 0.05), and whole-body velocities at peak whole-body power (1.03 ± 0.11 m/s vs. 0.69 ± 0.15 m/s, p < 0.05), respectively (Table 3).

Statistically significant differences were also detected in GRFs applied to the feet at the starting position and mean GRFs applied to the feet during the push-up. Females had larger GRFs applied to the feet at the starting position than males (0.36 ± 0.04 body weight vs. 0.33 ± 0.03 body weight, p < 0.05). Moreover, females also had larger mean GRFs applied to the feet during the push-up (0.33 ± 0.04 body weight vs. 0.28 ± 0.03 body weight, p < 0.05).

## Discussion

The purpose of the current study was to compare the force, velocity, and power parameters between the one-force-platform and two-force-platform methods during plyometric push-ups. The findings support the hypotheses that the two-force-platform and one-force-platform methods would demonstrate significant differences in all whole-body velocity and power parameters due to the non-constant force applied to the feet. Specifically, the average force applied to the feet throughout the push-up was smaller than its initial value at the starting position. Therefore, the assumption of a constant feet force overestimated the force and impulse applied to the feet throughout the push-up. Accumulatively, the one-force-platform method overestimated the peak whole-body velocities and power.

Consistent with the findings of previous studies [33, 34], the percentages of body weight supported by the hands were approximately 66%, while the force applied to the feet was close to 34% of the body weight at the starting position. As shown in Fig. 2, the forces applied to the feet were less than the initial value during most of the time of the push-up. While the force applied to the feet increased towards the end of the push-up, the average force applied to the feet throughout the push-up was only 30% of the body weight. During a push-up, the center of mass (COM) position shifted horizontally and vertically. The trajectory of the COM of the body is a circular arc around the fixed toes. Consistent with previous studies, as participants descended, COM shifted forward and downward with more loads being placed on arms, resulting in decreased forces placed on the feet. At the end of the push-up, COM shifted backward and upward and increased the forces applied to the feet [13, 32, 33]. Consequently, the two-force-platform and one-force-platform methods demonstrated similar peak whole-body forces and whole-body forces at the peak whole-body power, as these forces occurred at single time points during the later phase of the push-up. On the other hand, the movement velocity is calculated from the accumulative impulse from the starting position. The later increase in the forces applied to the feet could not compensate for the decreased forces during most of the phase and resulted in decreased whole-body velocities for the two-force-platform method. Power is the production of forces and velocities. The decreased whole-body velocity, rather than the whole-body force, was the main cause of decreased whole-body power. Consistent with previous studies [27, 28, 34], the peak force for men was 144% of body weight in the current study. Due to the differences in calculation methods and testing protocols, whole-body peak power from two force platforms was smaller than a previous study that used the one-force-platform method [24]. Meanwhile, males shifted more body weight toward hands during push-ups and demonstrated greater force, power, and velocity compared with females, highlighting the sex difference in strength, power, and motor control strategies [17, 35]. Secondary analyses revealed that the overall effect of calculation methods on the changes of force, power, and velocity were very similar between men and women. Thus, comparisons of the previous findings using the two-force-platform and one-force-platform methods for push-ups should be made with caution.

Despite the significant differences in most variables, the variables calculated from two-force-platform and one-force-platform methods significantly correlated during plyometric push-ups. While the two-force-platform method should be considered as the golden standard for plyometric push-ups, it might be challenging to implement two force platforms for testing during practical situations. When only one force platform is available, researchers and practitioners may calculate the whole-body velocities and power with the assumption of constant foot forces and then apply the linear regression equations reported in the current study to predict the more accurate whole-body velocities and power. It should also be noted that the prediction for the peak whole-body force was likely to be more accurate than the predictions for the peak whole-body velocity and power. In addition, the current regression equations are limited to the tested population with noticeable prediction errors for several variables.

Several limitations exist in the current study. First, no instructions regarding shoulder flexion angles, shoulder abduction angles, and other specific forms of push-ups were given and could introduce confounding factors in the current study. Second, the current study recruited physically active college-age students. Future investigations are needed to study other populations, such as highly trained or sedentary individuals. Third, only one variation of the plyometric push-up was included. Future studies may consider incorporating push-ups with other techniques. Fourth, the stretching protocol of the warm-up was not controlled. While a more controlled strengthen protocol might affect the magnitude of force production of the upper body, it was not likely to significantly affect the comparison between the two-force-platform and one-force-platform calculation methods for plyometric push-ups. Last, the number of participants was relatively small. Future efforts may need to recruit more participants to confirm outcomes from the current study.

## Conclusion

The one-force-platform method with the assumption of a constant force applied to the feet overestimated the whole-body velocities and power compared with the two-force-platform method during the plyometric push-up. These differences were mainly caused by decreased forces applied to the feet compared to the initial value throughout most of the push-up phase. Therefore, previous findings of push-up velocities and power using the two-force-platform and one-force-platform methods should be compared with caution. Using only one platform is a valid and reliable tool to assess force related variables produced by the hands, such as the peak forces, rate of force development, and impact forces of the hands [14, 33]. But it is logical to have inaccurate mechanical power outputs based on one platform under the hands because of the violation of conditions to use the impulse and momentum theorem. For sports scientists and professionals who need to accurately quantify kinetic variables during the plyometric push-up, the two-force-platform method should be considered. When only one force plate is available, linear regression equations may be used to predict velocities and power parameters obtain from one force platform.

## Data Availability

The datasets generated during and/or analyzed during the current study are not publicly available due to regulations in the Institutional Review Board, but may be made available from the corresponding author on reasonable request.
